# Concordance of Lung Cancer, Melanoma, and Renal Cell Cancer Diagnosis Information Recorded in Health Care Databases in England: Analysis of Linkage Between Primary Care, Hospital Care, and Cancer Registry Data

**DOI:** 10.1002/pds.70299

**Published:** 2026-01-14

**Authors:** Paul D. Kruithof, Patrick C. Souverein, Johanna H. M. Driessen, Lizza E. L. Hendriks, Sander Croes, Robin M. J. M. van Geel

**Affiliations:** ^1^ Department of Clinical Pharmacy & Toxicology CARIM, Research Institute for Cardiovascular Disease, Maastricht University Medical Centre+ Maastricht the Netherlands; ^2^ Division of Pharmacoepidemiology & Clinical Pharmacology Utrecht Institute for Pharmaceutical Sciences, Utrecht University Utrecht the Netherlands; ^3^ Department of Pulmonary Diseases GROW Research Institute for Oncology and Reproduction, Maastricht University Medical Center+ Maastricht the Netherlands

**Keywords:** concordance, electronic health records, lung cancer, melanoma, real‐world data, renal cell cancer, systemic anticancer treatments

## Abstract

**Purpose:**

Real‐world evidence (RWE) addresses clinical trial limitations by capturing more representative patient populations and improves evaluation of anticancer treatments, although it becomes available only years after market authorization. As many RWE sources capture only parts of the healthcare continuum, dataset linkage is necessary to enhance data richness. Linkage quality must be assessed to prevent information bias due to incomplete data linkage.

**Methods:**

We evaluated diagnosis concordance for lung cancer (LC), melanoma, and renal cell cancer (RCC) in England. Patients were matched based on national health service (NHS) number, sex and date of birth. Eligible patients were drawn from the National Cancer Registry and Analysis Service (NCRAS), and matched with three other datasets: Clinical Research Practice Database Aurum (CPRD Aurum), Hospital Episode Statistics Admitted Patient Care (HES‐APC), and systemic anticancer treatment (SACT). Concordance was evaluated for cancer diagnosis and date of diagnosis. Determinants of non‐concordance were investigated to assess representativeness.

**Results:**

In total, 89 797 patients with LC, melanoma or RCC were identified, and concordance of cancer diagnosis records between NCRAS, CPRD Aurum and HES‐APC exceeded 70%. Because patients are only registered in SACT upon receiving systemic anticancer treatment, matched numbers in SACT were significantly lower (3.0%–21.1%), as anticipated, particularly among patients over 80 years of age. However, differences in patient characteristics across datasets were limited. Concordance analyses showed that the majority of cases with LC diagnoses were registered within 3 months of the initial diagnosis within all data sources, whereas melanoma and RCC showed longer delays.

**Conclusions:**

Given the high concordance, NCRAS data can be enriched with HES‐APC and CPRD Aurum, and further complemented by SACT for systemic therapy. Provided that SACT undergoes further validation, linkage between NCRAS, CPRD Aurum, HES‐APC, and SACT may be a promising resource for RWE generation in oncology research.

## Key Points

- Registry linkage is essential for real‐world evidence, as key outcome measures are recorded in separate registries.
- NCRAS registrations of selected cancer diagnoses can be adequately linked with corresponding patient records in other datasets.
- Most lung cancer diagnoses were concordantly captured within 3 months of initial diagnosis.
- Melanoma and renal cell carcinoma diagnoses demonstrate longer registration delays.
- Provided SACT recording is validated, it may offer a promising opportunity for real‐world anticancer research, as over 90% of SACT‐registered patients can be linked to primary care data via CPRD Aurum.

## Plain Language Summary

RWE is important in the evaluation of outcomes of anticancer agents. Many RWE sources capture the healthcare continuum only partially, and linkage is needed to improve data richness. We evaluated diagnosis concordance in four datasets to assess linkage completeness. Patient records were drawn from the NCRAS if they had lung cancer, melanoma, or RCC between 2011 and 2018, and if linkage to CPRD Aurum was possible based on age, sex, and NHS number; 89797 patients in NCRAS were matched with data from CPRD Aurum, the HES‐APC, and SACT datasets. Diagnosis concordance was substantial between registries (all > 70%). Matched patients in SACT were limited, which seems consistent with the expected small number of cases eligible for and willing to receive systemic treatments. However, given the high concordance, NCRAS data can be enriched with HES‐APC and CPRD Aurum and further complemented by SACT data for systemic therapy. Provided that SACT undergoes further validation, linkage between NCRAS, CPRD Aurum, HES‐APC, and SACT may be a promising resource for RWE generation in oncology research.

**Abbreviations**
BMI: body mass index
BRAF: V‐Raf Murine Sarcoma Viral Oncogene Homolog B
CPRD: Clinical Practice Research Datalink
GP: general practitioner
HES‐APC: Hospital Episode Statistics Admitted Patient Care
LC: lung cancer
MEK: mitogen‐activated protein kinase
NCRAS: National Cancer Registry and Analysis Service
NHS: national health service
NSCLC: non‐small cell lung cancer
RCC: renal cell cancer
RWE: real‐world evidence
SACT: systemic anticancer treatment
SCLC: small cell lung cancer

## 1 Introduction

The demand for high‐quality real‐world evidence (RWE) to evaluate effectiveness of systemic anticancer therapies in daily clinical practice is rapidly increasing. Over the past decade, advancements in treatments, such as immunotherapies and targeted therapies, have significantly enhanced survival probabilities and quality of life for patients with cancer [1, 2]. Despite these improvements, clinical trials face persistent challenges, including patient enrollment difficulties and limited generalizability due to strict inclusion criteria. Patients with comorbidities are often excluded from clinical trials, whereas such conditions are common in daily practice [3]. Real‐world observational study designs offer a means to address these limitations, providing insights into more representative patient cohorts. However, many existing RWD sources capture only fragments of the healthcare continuum, like claims data, prescription records, or diagnostic codes [4]. This fragmentation increases the risk of information bias, particularly regarding comorbidities, adverse events, and mortality, which may only be recorded in specific care settings [5]. Integrating complementary datasets through data linkage could enhance data richness, but its quality must be rigorously evaluated to ensure the reliability and validity of the resulting analyses.

England offers some of the largest sources of RWD, with several linkage possibilities to a range of health care registries. Cancer diagnoses are primarily registered through the National Cancer Registry Analysis Service (NCRAS) [6]. Patients registered in NCRAS may be linked to external datasets, such as the clinical practice research database (CPRD) Aurum, which records information on primary care data, relying on information entered by general practitioners (GP), including demographics, prescription details, clinical events, preventive care provided, specialist referrals, hospital admissions and their major outcomes [7]. Similarly, patient records can be linked to the admitted patient care registry: Hospital Episode Statistics Admitted Patient Care (HES‐APC), containing information about any secondary care‐based activity requiring a hospital bed, including cancer drug therapies given at the oncology wards or daycare units [8, 9]. Furthermore, systemic treatment information is also captured in the systemic anticancer treatment (SACT) registries [10]. Data from these registries can be linked in order to provide a more comprehensive overview of healthcare provided in primary, secondary and tertiary care settings.

Previous linkage studies have primarily evaluated the concordance between the NCRAS, CPRD, and HES datasets, showing good overall linkage quality, including a recent study by Whitfield *et al.* [11, 12, 13] However, these analyses did not evaluate concordance between CPRD Aurum, which provides comprehensive longitudinal primary care data, and SACT, which uniquely captures systemic anticancer treatment information. Evaluating linkage between these datasets is therefore essential to enable studies that integrate detailed treatment information from SACT with real‐world outcomes recorded in CPRD Aurum. Establishing the feasibility and reliability of this linkage is a critical methodological step toward generating robust RWE on the effectiveness and safety of systemic anticancer therapies, thereby addressing a key gap in the current evidence base for evaluating these therapies in routine clinical practice. Therefore, in this study we assessed the concordance of the abovementioned registries in terms of cancer diagnosis and date of diagnosis for three cancer types, that is, lung cancer (LC), melanoma, and renal cell cancer (RCC), all of which were potentially eligible for systemic anticancer treatments during the study period, depending on factors such as disease stage. Finally, in order to investigate dataset representativeness and completeness, we investigated clinical determinants (i.e., sex, age, BMI, and smoking status) for dataset concordance.

## 2 Methods

### 2.1 National Cancer Registry and Analysis Service

For this study, NCRAS was used as a gold standard dataset, with which other datasets were compared. NCRAS records cancer data from multidisciplinary team meetings, pathology records, hospital administration systems and death records from the Office for National Statistics [6]. NCRAS' contribution is mandatory for all national health service (NHS) healthcare providers in England, thus the registry provides a high degree of coverage (estimated at 98%–99% of hospital activity in England) [6], with the remainder being primarily private hospitals, not funded by the NHS.

### 2.2 Clinical Practice Research Datalink

The CPRD Aurum (release February 2022: 13.3 million active participants) [14] comprises anonymized primary care medical records from enrolled GPs predominantly in England. This Aurum release covers data from around 41 million patients. It has been previously shown that the patient population covered in Aurum is representative of the broader population in terms of geographical spread, deprivation, age, and gender [7].

### 2.3 Hospital Episode Statistics Admitted Patient Care

HES‐APC data contains details of all admissions to, or attendances at English NHS healthcare providers. It includes patients treated in NHS hospitals, patients residing outside of England and care delivered by treatment centers (including those in the independent sector) funded by the NHS. All NHS healthcare providers in England provide data [8]. The exact degree of coverage is not reported; however, as the HES‐APC registry is tied to secondary care funding by the NHS, the degree of coverage is assumed similar to that of NCRAS (estimated at 98%–99% of hospital activity in England) [8]. Upon a patient's hospital discharge, a discharge summary is completed, which is then entered into the local patient information database based on a set of standardized rules [15, 16].

### 2.4 Systemic Anticancer Treatment Registry

The SACT dataset collects diagnostic and treatment‐related information on patients receiving systemic anticancer treatments, such as chemotherapy, immunotherapy, targeted therapies, endocrine therapies, and supportive treatments [10]. The dataset covers data on oncolytic medications prescribed to be delivered and (self)administered at the oncology day‐care center, at the ward, or at home. It is curated by the NCRAS registry, and data submission is mandatory for NHS health care providers (complete coverage of NHS trusts achieved in 2014) [10]. Data are extracted monthly from hospital electronic prescribing systems.

### 2.5 Study Population

The cohort was derived from a larger cohort study on type 1 diabetes mellitus incidence in patients with cancer. Consequently, patients with a diagnosis of type 1 diabetes before their cancer diagnosis are excluded from the cohort (*n* = 827, shown in Appendix 1). The study population consisted of all patients with a record for LC, melanoma or RCC in Aurum or NCRAS between July 1st 2011 and December 31st 2018. Cancer diagnoses were defined by SNOMED concept ID codes for Aurum, and International Classification of Diseases version 10 (ICD‐10) codes in HES‐APC, NCRAS or SACT (Appendix 2). Patients were included when they were research‐acceptable according to CPRD pre‐defined criteria, specifically having valid entries for year of birth, registration date, gender, health care episode dates, and if they had non‐temporary registration [17]. Patients were then linked between registries based on NHS number, name, date of birth and sex. Patients who could not be linked due to missing values in the matching variables were excluded. Differences in cancer diagnosis dates were allowed.

### 2.6 Assessments of Concordance

Patients with cancer identified in NCRAS were compared to diagnoses data from Aurum, HES‐APC, and SACT. For determining concordance, cancer cases in NCRAS were used as the source (reference) dataset. Records of cancer diagnoses were considered concordant if at least one confirmed diagnosis of the same cancer type (LC, melanoma, or RCC) was findable in both the source dataset as well as in the comparator dataset. The index date for each case was the date of first registered confirmed diagnosis for each dataset. Index dates in Aurum, HES‐APC, and SACT were compared to the index date in NCRAS for determining index date disparity between datasets. Cancer recurrence was not counted as a separate (new) diagnosis. If the cancer type did not match between the data source and comparator, or if the case was missing in the comparator, cases were classified as ‘non‐concordant’.

### 2.7 Data Analysis

Concordance was calculated as the percentage agreement in cancer diagnoses, through pairwise comparison. Concordance between datasets was expressed as a percentage of agreement. Differences between subgroups were explored to evaluate whether patient characteristics differed between datasets. Variables including sex, categorized age, ethnicity, categorized BMI, smoking history, index year and cancer stage were assessed using Pearson's Chi‐Square test, with a significance level of *p* < 0.05. All analyses were stratified by cancer type. Data analysis was performed using SAS v9.4, SAS Institute Inc., Cary, NC, USA. The manuscript was prepared in accordance with the Strengthening the Reporting of Observational Studies in Epidemiology (STROBE) statement (Appendix 2).

## 3 Results

A total of 89 797 patients with cancer were identified in NCRAS, comprising 56 408 patients with LC, 18926 patients with melanoma, and 14 463 patients with RCC (Table 1). In Aurum, the total number of patients with any of these three cancer types was slightly higher, at 92653. In HES‐APC, 77082 patients were identified, and in SACT, 12527 patients were recorded. Within SACT, 4 patients were registered with a second primary cancer type that was also included in the study cohort.

**TABLE 1:** Baseline characteristics of included patients from NCRAS, Aurum, HES‐APC, and SACT.

		NCRAS		Aurum		HES‐APC		SACT	
		*n*/mean	SD/%	*n*/mean	SD/%	*n*/mean	SD/%	*n*/mean	SD/%
Lung cancer									
	Patients	56 408		58 160		50 952		10 903	
	Mean follow‐up time (years)	0.5	±0.7	1.0	±1.4	0.8	±1.3	0.7	±1.0
	Gender								
	Female	26 386	(46.8%)	27 238	(46.8%)	23 744	(46.6%)	5060	(46.4%)
	Male	30 022	(53.2%)	30 922	(53.2%)	27 208	(53.4%)	5843	(53.6%)
	Age at index date								
	Mean age (years)	73.3	±10.9	72.7	±11.0	72.2	±10.9	68.0	±9.4
	18–49 years	1368	(2.4%)	1499	(2.6%)	1370	(2.7%)	441	(4.0%)
	50–59 years	5017	(8.9%)	5283	(9.1%)	4920	(9.7%)	1630	(14.9%)
	60–69 years	14 129	(25.0%)	14 586	(25.1%)	13 471	(26.4%)	3973	(36.4%)
	70–79 years	19 450	(34.5%)	19 972	(34.3%)	17 786	(34.9%)	3947	(36.1%)
	80+ years	16 444	(29.2%)	16 820	(28.9%)	13 405	(26.3%)	912	(8.4%)
Melanoma									
	Patients	18 926		19 391		13 891		593	
	Mean follow‐up time (years)	0.6	±1.0	3.0	±2.1	2.1	±2.1	1.0	±1.1
	Gender								
	Female	9627	(50.9%)	9940	(51.3%)	7109	(51.2%)	218	(36.8%)
	Male	9299	(49.1%)	9451	(48.7%)	6782	(48.8%)	375	(63.2%)
	Age at index date								
	Mean age (years)	63.3	±16.7	62.4	±16.8	63.1	±16.5	65.4	±14.3
	18–49 years	4372	(23.1%)	4629	(23.9%)	3101	(22.3%)	87	(14.6%)
	50–59 years	3175	(16.8%)	3287	(17.0%)	2336	(16.8%)	99	(16.6%)
	60–69 years	4051	(21.4%)	4158	(21.4%)	3058	(22.0%)	161	(27.0%)
	70–79 years	4048	(21.4%)	4092	(21.1%)	3065	(22.1%)	165	(27.6%)
	80+ years	3280	(17.3%)	3225	(16.6%)	2331	(16.8%)	81	(13.6%)
Renal cell cancer									
	Patients	14 463		15 102		12 239		1017	
	Mean follow‐up time (years)	0.6	±0.9	2.3	±2.0	1.8	±2.0	1.2	±1.5
	Gender								
	Female	5312	(36.7%)	5578	(36.9%)	4474	(36.6%)	330	(32.4%)
	Male	9151	(63.3%)	9524	(63.1%)	7765	(63.4%)	687	(67.6%)
	Age at index date								
	Mean age (years)	69.1	±13.2	68.6	±13.2	67.7	±13.1	65.9	±11.0
	18–49 years	1300	(9.0%)	1361	(9.0%)	1188	(9.7%)	85	(8.3%)
	50–59 years	2234	(15.4%)	2305	(15.3%)	2044	(16.7%)	204	(20.0%)
	60–69 years	3618	(25.0%)	3764	(24.9%)	3252	(26.6%)	325	(31.9%)
	70–79 years	4106	(28.4%)	4324	(28.6%)	3462	(28.3%)	324	(31.8%)
	80+ years	3205	(22.2%)	3348	(22.2%)	2293	(18.7%)	79	(7.8%)

Abbreviations: CPRD: clinical practice research database; HES‐APC: Hospital Episode Statistics Admitted Patient Care; NCRAS: National Cancer Registry Analysis Service; SACT: systemic anticancer treatment.

Baseline characteristics for LC and RCC were generally similar across datasets in terms of age and sex distribution (Table 1). Median duration of follow‐up was longest in primary care data (LC: 1.0 years; melanoma: 3.0 years; RCC: 2.3 years) and shortest in NCRAS (0.5–0.6 years). In SACT, median follow‐up duration was 0.7, 1.0, and 1.2 years for LC, melanoma, and RCC, respectively. Two notable differences at baseline were a lower proportion of female patients with melanoma in SACT (36.8%) compared with NCRAS (50.9%), and a lower representation of patients aged over 80 years for RCC and melanoma in SACT.

NCRAS and Aurum demonstrated high agreement for cancer diagnoses, exceeding 90% for all cancer types (Table 2). Interestingly, for all three cancer types we found diagnoses in Aurum (4%–6% of Aurum cases) which could not be confirmed using NCRAS. Nearly all cases identified in HES‐APC were also present in NCRAS and Aurum (> 95% for all cancer types). However, the reverse was not true: between 71.6% and 87.6% of cases recorded in NCRAS and Aurum were also identifiable in HES‐APC. In SACT, most registered cases were also found in the other datasets (> 92% agreement), although the proportion of all cancer patients with a record in SACT was lower, ranging from 18.6%–21.1% for LC, 6.6%–7.9% for RCC, and 3.0%–4.0% for melanoma.

**TABLE 2:** Pairwise comparison of cancer diagnoses between NCRAS, CPRD Aurum, HES‐APC, NCRAS, and SACT.

Source database	Primary cancer diagnosis	Cases in source database	Comparator databases			
			NCRAS	Aurum	HES‐APC	SACT
			*n* (% of ref.)	*n* (% of ref.)	*n* (% of ref.)	*n* (% of ref.)
NCRAS	Lung cancer	56 408	—	55 145 (97.8%)	48 935 (86.8%)	10 868 (19.3%)
	Melanoma	18 926	—	18 555 (98.0%)	13 630 (72.0%)	589 (3.1%)
	Renal cell cancer	14 463	—	14 163 (97.9%)	11 808 (81.6%)	1009 (7.0%)
Aurum	Lung cancer	58 160	55 145 (94.8%)	—	50 952 (87.6%)	10 843 (18.6%)
	Melanoma	19 391	18 555 (95.7%)	—	13 891 (71.6%)	586 (3.0%)
	Renal cell cancer	15 102	14 163 (93.8%)	—	12 239 (81.0%)	1003 (6.6%)
HES‐APC	Lung cancer	50 952	48 935 (96.0%)	50 952 (100%)	—	10 745 (21.1%)
	Melanoma	13 891	13 630 (98.1%)	13 891 (100%)	—	550 (4.0%)
	Renal cell cancer	12 239	11 808 (96.5%)	12 239 (100%)	—	966 (7.9%)
SACT	Lung cancer	10 920	10 868 (99.5%)	10 843 (99.3%)	10 745 (98.4%)	—
	Melanoma	597	589 (98.7%)	586 (98.2%)	550 (92.1%)	—
	Renal cell cancer	1018	1009 (99.1%)	1003 (98.5%)	966 (94.9%)	—

Abbreviations: HES‐APC: Hospital Episode Statistics Admitted Patient Care; NCRAS: National Cancer Registry Analysis Service; SACT: Systemic Anticancer Treatment.

The timing of diagnosis recording for concordantly registered patients is shown in Table 3. For Aurum and HES‐APC, the majority of cases were recorded in both NCRAS and the comparator dataset within 0–3 months of the NCRAS diagnosis date. In SACT, most LC cases were also recorded within this time frame; however, a substantial proportion of melanoma (87.5%) and RCC (58.3%) cases were registered more than 3 months after the initial diagnosis date.

**TABLE 3:** Index date disparity of cancer diagnoses found in NCRAS as compared to CPRD Aurum, HES‐APC, and SACT.

Comparator	Diagnosis	Concordant cases	Within 1 month		Within 1–3 months		Within 3–12 months		After 1+ year	
		*n*	*n* (% of source)		*n* (% of source)		*n* (% of source)		*n* (% of source)	
Aurum	LC	55 145	47 871	(86.8%)	5731	(10.4%)	1239	(2.2%)	304	(0.6%)
	Melanoma	18 555	17 989	(96.9%)	329	(1.8%)	146	(0.8%)	91	(0.5%)
	RCC	14 163	10 365	(73.2%)	3071	(21.7%)	654	(4.6%)	73	(0.5%)
HES‐APC	LC	48 935	34 169	(69.8%)	9540	(19.5%)	3900	(8.0%)	1326	(2.7%)
	Melanoma	13 630	6380	(46.8%)	5550	(40.7%)	1041	(7.6%)	659	(4.8%)
	RCC	11 808	5965	(50.5%)	3671	(31.1%)	1583	(13.4%)	589	(5.0%)
SACT	LC	10 868	2558	(23.5%)	5785	(53.2%)	1966	(18.1%)	559	(5.1%)
	Melanoma	589	15	(2.5%)	59	(10.0%)	172	(29.2%)	343	(58.2%)
	RCC	1009	111	(11.0%)	310	(30.7%)	358	(35.5%)	230	(22.8%)

Abbreviations: HES‐APC: Hospital Episode Statistics Admitted Patient Care; LC: lung cancer; NCRAS: National Cancer Registry Analysis Service; RCC: renal cell cancer; SACT: systemic anticancer treatment.

Diagnosis record concordance by patient characteristics was compared with the cases in the NCRAS registry (Table 4). Male patients were slightly more likely to have concordant registration with the reference NCRAS dataset. Age also influenced concordance: in HES‐APC and SACT, LC and RCC patients aged 80 years or older had lower agreement (all *p* < 0.001). In SACT, concordance increased between 2011 and 2014 (all *p* < 0.001, sensitivity analysis shown in Appendix 3 (Table A1)). For melanoma and RCC in HES‐APC and SACT, concordance declined towards the end of the study period.

**TABLE 4:** Linkage rate by clinical determinant between cases found within the comparator datasets in relation to the cases in the NCRAS registry, stratified by cancer diagnosis. Differences between subgroups were assessed using Pearson's Chi‐square method.

	Lung cancer			Melanoma			Renal Cell cancer		
	Aurum	HES‐APC	SACT	Aurum	HES‐APC	SACT	Aurum	HES‐APC	SACT
Sex^A^									
Female	100%	88.3%	19.5%	100%	73.7%	2.3%	100%	83.1%	6.2%
Male	100%	89.1%	19.8%	100%	73.3%	4.0%	100%	83.5%	7.6%
Χ^2^ (df = 1)	—	8.61	0.81	—	0.36	45.63	—	0.42	9.47
*p* value	—	0.003	0.367	—	0.549	< 0.001	—	0.517	0.002
Age at diagnosis^A^									
< 50 years	100%	93.5%	33.9%	100%	70.2%	2.5%	100%	88.8%	7.1%
50–59 years	100%	94.0%	33.5%	100%	72.5%	3.2%	100%	90.4%	9.7%
60–69 years	100%	93.3%	28.8%	100%	75.0%	4.1%	100%	87.9%	9.4%
70–79 years	100%	90.3%	20.2%	100%	75.6%	3.6%	100%	83.1%	7.5%
80+ years	100%	80.8%	5.4%	100%	74.2%	2.1%	100%	71.0%	2.0%
*Χ* ^2^ (df = 4)	—	1495.20	3564.32	—	40.20	32.34	—	494.74	173.73
*p* value	—	< 0.001	< 0.001	—	< 0.001	< 0.001	—	< 0.001	< 0.001
Ethnicity^B^									
Asian	100%	100%	26.0%	100%	100%	4.9%	100%	100%	7.6%
Black	100%	100%	27.3%	100%	100%	6.3%	100%	100%	8.4%
White	100%	100%	21.7%	100%	100%	4.1%	100%	100%	8.2%
Not reported	100%	100%	21.6%	100%	100%	0%	100%	100%	7.2%
*Χ* ^2^ (df = 3)	—	—	26.51	—	—	19.07	—	—	0.65
*p* value	—	—	< 0.001	—	—	< 0.001	—	—	0.886
BMI at diagnosis^C^									
< 20 kg/m^2^	100%	83.4%	12.0%	100%	71.5%	2.0%	100%	77.3%	6.8%
20–24.9 kg/m^2^	100%	88.2%	19.3%	100%	72.3%	3.1%	100%	80.8%	7.0%
25–29.9 kg/m^2^	100%	90.3%	21.5%	100%	73.3%	3.1%	100%	83.9%	7.4%
30–34.9 kg/m^2^	100%	91.1%	22.2%	100%	75.2%	3.5%	100%	84.9%	7.2%
35+ kg/m^2^	100%	90.0%	21.1%	100%	76.5%	3.2%	100%	86.7%	6.1%
Not reported	100%	85.5%	16.7%	100%	72.8%	3.3%	100%	82.0%	7.1%
*Χ* ^2^ (df = 5)	—	294.46	302.40	—	16.68	4.80	—	49.21	3.23
*p* value	—	< 0.001	< 0.001	—	0.005	0.441	—	< 0.001	0.664
Smoking history^C^									
Never	100%	86.2%	17.3%	100%	73.6%	3.1%	100%	82.4%	6.5%
Current	100%	90.1%	21.4%	100%	74.8%	3.6%	100%	86.0%	7.9%
Former	100%	87.2%	16.7%	100%	71.0%	3.0%	100%	81.4%	6.6%
Not reported	100%	86.0%	15.4%	100%	75.1%	2.6%	100%	81.7%	9.6%
*Χ* ^2^ (df = 3)	—	158.62	174.38	—	14.47	4.20	—	35.41	13.54
*p* value	—	< 0.001	< 0.001	—	0.002	0.241	—	< 0.001	0.003
Diagnosis year^D^									
2011	97.9%	88.3%	3.8%	99.01%	72.5%	2.2%	97.58%	82.9%	3.1%
2012	97.3%	87.5%	11.1%	98.66%	71.1%	2.7%	97.13%	84.0%	5.6%
2013	97.3%	87.1%	15.2%	98.25%	70.0%	2.8%	97.16%	82.0%	5.9%
2014	98.0%	87.7%	21.6%	97.80%	72.6%	3.8%	98.56%	83.3%	7.5%
2015	98.1%	87.9%	23.5%	97.60%	73.2%	4.2%	98.12%	83.5%	8.2%
2016	98.2%	88.0%	24.9%	98.31%	74.6%	3.3%	98.12%	82.9%	8.8%
2017	97.9%	86.4%	25.5%	97.76%	74.9%	3.6%	98.61%	82.6%	8.3%
2018	97.6%	82.0%	20.6%	97.61%	67.7%	2.0%	97.77%	72.6%	5.7%
*Χ* ^2^ (df = 7)	28.79	182.24	1435.43	18.43	55.34	33.13	21.24	121.99	53.33
*p* value	< 0.001	< 0.001	< 0.001	0.010	< 0.001	< 0.001	0.003	< 0.001	< 0.001
Cancer stage at diagnosis^E^									
In situ	99.7%	98.5%	100%	100%	80.8%	100%	100%	96.9%	100%
Stage I	99.2%	83.9%	100%	98.77%	88.9%	100%	100%	95.4%	100%
Stage II	99.5%	99.4%	100%	100%	87.5%	100%	100%	94.0%	100%
Stage III	99.6%	98.3%	100%	100%	91.7%	100%	100%	92.9%	100%
Stage IV	99.7%	98.9%	100%	99.17%	95.8%	100%	99.00%	96.2%	100%
Not reported	97.3%	99.0%	0.2%	98.00%	71.4%	0.1%	97.82%	80.6%	0.0%
*Χ* ^2^ (df = 5)	207.46	1670.99	56 408	5.72	139.95	18 926	11.22	144.53	14 463
*p* value	< 0.001	< 0.001	< 0.001	0.456	< 0.001	< 0.001	0.082	< 0.001	< 0.001

Abbreviations: BMI: body mass index; CPRD: clinical practice research database; HES‐APC: Hospital Episode Statistics Admitted Patient Care; NCRAS: National Cancer Registry Analysis Service; SACT: systemic anticancer treatment.

^A^ (Derived from a) matching variable.

^B^ Information available in NCRAS, HES‐APC, and Aurum.

^C^ Information available only in Aurum.

^D^ Information available in all datasets.

^E^ Information available in SACT.

## 4 Discussion

In this study, we evaluated the concordance of cancer diagnosis capturing between four electronic health registries with NCRAS as reference. Baseline characteristics were generally consistent between datasets for all evaluated cancer types, although SACT contained fewer linkable women than men with melanoma and fewer linkable patients aged 80 years or older compared to younger age categories for LC and RCC. The latter likely reflects the greater vulnerability of patients over 80 years of age who are generally less eligible for systemic anticancer treatment [10, 18, 19]. The available duration of patient follow‐up was longest in primary care (CPRD Aurum) and shortest in NCRAS. Concordance of cancer diagnosis between CPRD Aurum and NCRAS exceeded 90% for all cancer types, and the majority of HES‐APC cases were also present in these datasets. We found that 4%–6% of patients who had a diagnosis in CPRD Aurum did not have a concurrent diagnosis in NCRAS, which is considered the gold standard reference. A robust explanation for this could not be found within the available data. It is possible that some Aurum‐linked general practitioners register suspected, unconfirmed diagnoses, that patients become lost to follow‐up (e.g., due to death before referral or patient migration), or that patients have opted for private, non‐NHS funded healthcare more frequently than previously expected (approximately 1%–2%) [6].

SACT included a limited part of NCRAS LC, melanoma and RCC diagnoses, reflecting that not all patients received systemic anticancer therapies. The captured proportion was consistent with the number of patients reported to receive such treatments in England before 2018. For example, one study reported that approximately 33% of patients with advanced non‐small cell lung cancer (NSCLC) received systemic therapy in a similar study period [20]. In contrast, we found that 19% of all LC patients found in NCRAS were also registered in SACT. This lower proportion likely reflects the differences in study populations: the NCRAS registry includes patients not eligible for systemic therapy under clinical guidelines, such as those with very early‐stage NSCLC. Given that about one‐third of NSCLC patients are generally not eligible for systemic therapy based on disease stage [20], our 19% coverage corresponds to roughly 28% of treatment‐eligible patients. Thus, although SACT coverage appears limited at first glance, it likely reflects the broader inclusion criteria of our dataset rather than incomplete data capture. In contrast to LC, less than 8% of melanoma patients present with advanced disease at diagnosis [21]. As patients with resectable melanoma are generally treated in outpatient care (e.g., dermatological excisions) [22], relatively few melanoma cases were captured in SACT. Although access to systemic treatments expanded during the study period, initially with ipilimumab (2012) [23] and later with targeted therapies such as vemurafenib and cobimetinib for *BRAF* V600 mutated melanoma (2016) [24], this did not translate into a clear increase in melanoma registrations in SACT. This may partially be due to orally administered TKIs being more poorly recorded in SACT [11, 12, 25]. RCC generally presents with a less aggressive disease course compared to LC and melanoma, with longer survival reported for patients with stages I‐III [21], who therefore are less likely to require systemic therapy. However, patients diagnosed with stage 4 RCC between 2012 and 2014 often received systemic treatment [21]. Consistent with our expectations based on aforementioned literature, only 7% of patients could be matched with a diagnosis of RCC in NCRAS, compared to 19.3% for patients with LC.

Furthermore, to establish SACT as a reliable source of RWE, additional research is required to evaluate and validate the completeness and adequateness of data captured in SACT in more recent study periods. For patients included in SACT, cancer diagnosis concordance with the other datasets was high. Most cases were recorded within 3 months of diagnosis in CPRD Aurum, HES‐APC, and for LC in SACT; however, melanoma and RCC registrations in SACT showed more frequent recording delays. Similarly, year of diagnosis could affect data completeness, as SACT diagnosis concordance improved noticeably from 2014 onward when reporting became mandatory for all NHS trusts [10]. Minor declines in melanoma and RCC recording near the end of the study period are probably due to registration delays, as seen in Table 3.

The concordance of CPRD primary care data with HES‐APC and NCRAS has been previously assessed for the 20 most commonly found cancer sites, including LC, RCC, and melanoma [26]. Furthermore, several studies evaluated data quality of SACT and linkage to NCRAS for breast cancer and colorectal cancer [11, 12, 25]. Recently, Whitfield et al. [13] studied concordance on diagnosis reported across different datasets for several malignancies, among which LC. They found 84%–92% concordance of diagnoses between the separate registries, which is largely similar to the percentage of linkable cases we found for LC and RCC. However, a direct assessment of concordance between CPRD Aurum and SACT is lacking. We therefore evaluated the feasibility of linking cancer patients within SACT to CPRD Aurum, using NCRAS as gold standard. This potentially enables combined analyses of systemic treatment data extracted from SACT and real‐world outcome data retrieved from CPRD Aurum. This approach, to the best of our knowledge, has not been explored previously and addresses a critical gap in the potential use of these databases for the outcome evaluation of anticancer treatments.

The strengths of this study are twofold. First, we demonstrated the feasibility of linking SACT records with CPRD Aurum data through NCRAS, which served as the reference dataset. Second, this multi‐source linkage enables longitudinal follow‐up of patients in primary care (CPRD Aurum), extending observation time beyond what would be possible solely with SACT or NCRAS. This study has several limitations. First, we excluded patients with a record of type 1 diabetes mellitus prior to their cancer diagnosis. As the prevalence of type 1 diabetes is low (i.e., < 0.5%, according to the NHS) [27] and it is not generally associated with any of the studied cancer types, we do not believe this exclusion has meaningfully influenced our results. Second, in the HES registry, we only had access to admitted patient care (APC) data, but not to outpatient care data (OP). Therefore, it is likely that our study suffered from some underregistration with regard to patient diagnoses, oral anticancer treatment and outcomes in HES data. This could help explain why the concordance for HES‐APC with NCRAS as reference is lower (72.0%–86.8% agreement) compared to vice versa (96.5%–98.1% agreement). Finally, because we did not evaluate the correctness and completeness of the anticancer agent data reported in SACT, we could not draw strong conclusions on the feasibility of using SACT for RWE research and further research is needed to assess the validity of SACT capturing in more recent periods.

RWE can help to overcome clinical trial limitations by capturing more representative patient populations. Assuming sufficient coverage of SACT, linkage to Aurum, HES‐APC and SACT has the potential to support the evaluation of new anticancer treatments, although RWE becomes only available some years after market authorization. When using the SACT database, it is advisable to complement the data with hospital data linkage (both inpatient and outpatient), starting from 2014 onwards. Adding drug dosage and cycle information may support time‐ and dose‐dependent analyses of drug effectiveness and toxicity, while including performance status and cancer staging is valuable for real‐world analyses.

## 5 Conclusion

Key information required to conduct real‐world studies may be distributed across different data sources, underscoring the importance of registry linkage for generating robust real‐world evidence. Our findings demonstrate that NCRAS registrations of selected cancer diagnoses can be adequately linked with corresponding patient records in other datasets. Provided that SACT undergoes validation, SACT linkage may be a promising resource for RWE generation in oncology research, as over 90% of SACT‐registered patients can be linked to NCRAS, HES‐APC, and CPRD Aurum.

## Funding

The authors have nothing to report.

## Conflicts of Interest

Lizza Hendriks—Outside of this manuscript: research funding Roche Genentech, AstraZeneca, Boehringer Ingelheim, Takeda, Merck, Pfizer, Novartis, Gilead, all to institution. Summit under negotiation (EORTC), Amgen under negotiation. Speaker educationals/webinars: AstraZeneca, Bayer, Lilly, MSD, high5oncology, Takeda, Janssen, GSK, Sanofi, Pfizer (Inst), Medtalks, Benecke, VJOncology, Medimix (self). Advisory boards: Abbvie, Amgen, Anhearth, AstraZeneca, Bayer, BMS, Boehringer Ingelheim, Daiichi, GSK, Gilead, Janssen, Lilly, Merck, MSD, Novartis, Pfizer, Pierre Fabre, Roche, Sanofi, Summit Therapeutics, Takeda (all to institution). Member guideline committees: Dutch guidelines on NSCLC, brain metastases and leptomeningeal metastases (payment to self), ESMO guidelines on metastatic NSCLC, non‐metastatic NSCLC and SCLC (non‐financial). Other (non‐financial): former secretary and current chair NVALT studies foundation, former subchair and current secretary EORTC metastatic NSCLC systemic therapy, vice‐chair scientific committee Dutch Thoracic Group. Local PI of clinical trials: AstraZeneca, GSK, Novartis, Merck, Roche, Takeda, Blueprint, Mirati, Abbvie, Gilead, MSD, Merck, Amgen, Boehringer Ingelheim, Pfizer, Daiichi, Amgen (all to institution). The other authors declare no conflicts of interest.

## Data Availability

This study is based in part on data from the Clinical Practice Research Datalink obtained under licence from the UK Medicines and Healthcare products Regulatory Agency. The data is provided by patients and collected by the NHS as part of their care and support. The interpretation and conclusions contained in this study are those of the authors' alone. The data underlying this article were obtained through a multi‐study institutional license and are not publicly available. Details on how to apply for data access can be found at https://cprd.com/data‐access. Hospital Episode Statistics (HES) and NCRAS data Copyright (2023), re‐used with the permission of The Health & Social Care Information Centre. All rights reserved. The protocol for this research was approved by CPRD's Research Data Governance (RDG) Process (protocol 21_000413).

## Appendix 1 Data Delivery Letter

**CPRD Aurum**

**Cohort definition in CPRD Aurum**.

- Acceptable patients
- *Male/female* patients
- Patients eligible for linkage to HES and NCRAS data
- Patients were registered during the study period of 01/07/2011–31/12/2018

From the source population in CPRD Aurum:

- Inclusions
  ○ Patients had a record of lung cancer, melanoma, or renal cell carcinoma defined as at least:
    - one observation event in CPRD Aurum (as defined by the code list detailed in Appendices 8–10) or
    - an ICD‐10 code in the site_icd10_o2 variable in the NCRAS Cancer Registration file (as defined by the code list detailed in Appendix 4–6)
  ○ Patients had their first ever record for lung cancer, melanoma or renal cell carcinoma within the study period of 01/07/2011–31/12/2018
  ○ Patients had their index date within their follow up period.
  ○ Patients were aged 18 or older on their index date.
- Exclusions
  ○ Patients had a record of TIDM any time prior to their index date in their observation file (as defined by the code list detailed in Appendix 8).

**Data delivery**

- 40,933,535 acceptable patients
- 40,932,007 *male/female* patients
- 35,088,002 patients eligible for linkage to HES and NCRAS data
- 20,881,078 patients were registered during the study period of 01/07/2011–31/12/2018

20,881,078 are in the CPRD Aurum source population.

From the source population in CPRD Aurum:

- Inclusions
  ○ 292,497 patients had a record of lung cancer, melanoma, or renal cell carcinoma defined as at least:
    - one observation event in CPRD Aurum (as defined by the code list detailed in Appendices 8–10) or
    - an ICD‐10 code in the site_icd10_o2 variable in the NCRAS Cancer Registration file (as defined by the code list detailed in Appendices 4–6)
  ○ 149,587 patients had their first ever record for lung cancer, melanoma or renal cell carcinoma within the study period of 01/07/2011–31/12/2018
  ○ 121,172 patients had their index date within their follow up period.
  ○ 120,286 patients were aged 18 or older on their index date.
- Exclusions
  ○ 890 patients had a record of TIDM any time prior to their index date in their observation file (as defined by the code list detailed in Appendix 11)

There were 119,396 patients in the CPRD Aurum Cohort.

## Appendix 2 STROBE Checklist

STROBE Statement—checklist of items that should be included in reports of observational studies.

**Table:**

	Item No.	Recommendation	Page No.	Relevant text from manuscript
Title and abstract	1	(a) Indicate the study's design with a commonly used term in the title or the abstract	1	“Concordance”
		(b) Provide in the abstract an informative and balanced summary of what was done and what was found	5	“We evaluated diagnosis concordance […]” “In total, 89,797 patients with LC, melanoma or RCC were identified, and concordance of cancer diagnosis records between NCRAS, CPRD Aurum and HES‐APC exceeded 70% […]”
Introduction				
Background/rationale	2	Explain the scientific background and rationale for the investigation being reported	6	“Evaluating linkage between these datasets is therefore essential to enable studies that integrate detailed treatment information from SACT with real‐world outcomes recorded in CPRD Aurum.”
Objectives	3	State specific objectives, including any prespecified hypotheses	7	“Therefore, in this study we assessed the concordance of the abovementioned registries in terms of cancer diagnosis and date of diagnosis for three cancer types […]”
Methods				
Study design	4	Present key elements of study design early in the paper	8	“Patients with cancer identified in NCRAS were compared to diagnoses data from Aurum, HES‐APC and SACT. For determining concordance, […]”
Setting	5	Describe the setting, locations, and relevant dates, including periods of recruitment, exposure, follow‐up, and data collection	8	“The cohort was derived from […]”
Participants	6	(a) *Cohort study*—Give the eligibility criteria, and the sources and methods of selection of participants. Describe methods of follow‐up *Case‐control study*—Give the eligibility criteria, and the sources and methods of case ascertainment and control selection. Give the rationale for the choice of cases and controls *Cross‐sectional study*—Give the eligibility criteria, and the sources and methods of selection of participants	8	“The study population consisted of all patients with a record for […]”
		(b) *Cohort study*—For matched studies, give matching criteria and number of exposed and unexposed *Case‐control study*—For matched studies, give matching criteria and the number of controls per case	—	—
Variables	7	Clearly define all outcomes, exposures, predictors, potential confounders, and effect modifiers. Give diagnostic criteria, if applicable	8	“Concordance was calculated as the percentage agreement in cancer diagnoses, through pairwise comparison. Concordance between datasets was expressed as percentage of agreement. […]”
Data sources/measurement	8^a^	For each variable of interest, give sources of data and details of methods of assessment (measurement). Describe comparability of assessment methods if there is more than one group	8	
Bias	9	Describe any efforts to address potential sources of bias	—	
Study size	10	Explain how the study size was arrived at	—	
Quantitative variables	11	Explain how quantitative variables were handled in the analyses. If applicable, describe which groupings were chosen and why	—	
Statistical methods	12	(*a*) Describe all statistical methods, including those used to control for confounding	8	“[…] were assessed using Pearson's Chi‐Square test, with a significance level of *p* < 0.05 […]”
		(*b*) Describe any methods used to examine subgroups and interactions	—	
		(*c*) Explain how missing data were addressed	—	
		(*d*) *Cohort study*—If applicable, explain how loss to follow‐up was addressed *Case‐control study*—If applicable, explain how matching of cases and controls was addressed *Cross‐sectional study*—If applicable, describe analytical methods taking account of sampling strategy	—	
		(*e*) Describe any sensitivity analyses	9	“In SACT, concordance increased between 2011 and 2014 (all *p* < 0.001, sensitivity analysis shown in Appendix 3).”
Results				
Participants	13^a^	(a) Report numbers of individuals at each stage of study—e.g., numbers potentially eligible, examined for eligibility, confirmed eligible, included in the study, completing follow‐up, and analyzed	Appendix 1	
		(b) Give reasons for non‐participation at each stage	Appendix 1	
		(c) Consider use of a flow diagram	—	
Descriptive data	14^a^	(a) Give characteristics of study participants (e.g., demographic, clinical, social) and information on exposures and potential confounders	16	“Table 1. Baseline characteristics of included patients from NCRAS, Aurum, HES‐APC, and SACT”
		(b) Indicate number of participants with missing data for each variable of interest	—	
		(c) *Cohort study*—Summarize follow‐up time (e.g., average and total amount)	—	—
Outcome data	15^a^	*Cohort study*—Report numbers of outcome events or summary measures over time	17	“Table 2. Pairwise comparison of cancer diagnoses between NCRAS, CPRD Aurum, HES‐APC, NCRAS and SACT”
		*Case‐control study*—Report numbers in each exposure category, or summary measures of exposure	—	—
		*Cross‐sectional study*—Report numbers of outcome events or summary measures	—	—
Main results	16	(*a*) Give unadjusted estimates and, if applicable, confounder‐adjusted estimates and their precision (e.g., 95% confidence interval). Make clear which confounders were adjusted for and why they were included	18	Table 4.
		(*b*) Report category boundaries when continuous variables were categorized	18	Table 4.
		(*c*) If relevant, consider translating estimates of relative risk into absolute risk for a meaningful time period	—	—
Other analyses	17	Report other analyses done—e.g., analyses of subgroups and interactions, and sensitivity analyses	Appendix 3	
Discussion				
Key results	18	Summarize key results with reference to study objectives	10	“In this study, we evaluated concordance of cancer diagnosis […]”
Limitations	19	Discuss limitations of the study, taking into account sources of potential bias or imprecision. Discuss both direction and magnitude of any potential bias	11	“This study has several limitations. First, […]”
Interpretation	20	Give a cautious overall interpretation of results considering objectives, limitations, multiplicity of analyses, results from similar studies, and other relevant evidence	10	“SACT included a limited part of […]”
Generalizability	21	Discuss the generalizability (external validity) of the study results	—	
Other information				
Funding	22	Give the source of funding and the role of the funders for the present study and, if applicable, for the original study on which the present article is based	—	

*Note:* An Explanation and Elaboration article discusses each checklist item and gives methodological background and published examples of transparent reporting. The STROBE checklist is best used in conjunction with this article (freely available on the Web sites of PLoS Medicine at http://www.plosmedicine.org/, Annals of Internal Medicine at http://www.annals.org/, and Epidemiology at http://www.epidem.com/). Information on the STROBE Initiative is available at www.strobe‐statement.org.

^a^ Give information separately for cases and controls in case‐control studies and, if applicable, for exposed and unexposed groups in cohort and cross‐sectional studies.

## Appendix 3 Concordance From 2014 to 2018

**TABLE A1:** Pairwise comparison of cancer diagnoses between 2014 and 2018 in NCRAS, CPRD Aurum, HES‐APC, NCRAS, and SACT.

Source database	Primary cancer diagnosis	Cases in source database	Comparator databases			
			NCRAS *n* (% of ref.)	Aurum *n* (% of ref.)	HES APC *n* (% of ref.)	SACT *n* (% of ref.)
NCRAS	Lung cancer	37384	—	36612 (97.9%)	32287 (86.4%)	8708 (23.3%)
	Melanoma	13016	—	12727 (97.8%)	9384 (72.1%)	436 (3.3%)
	Renal cell cancer	9849	—	9674 (98.2%)	7965 (80.9%)	764 (7.8%)
Aurum	Lung cancer	38608	36612 (94.8%)	—	33571 (87.0%)	8672 (22.5%)
	Melanoma	17027	12727 (74.7%)	—	11010 (64.7%)	453 (2.7%)
	Renal cell cancer	10311	9674 (93.8%)	—	8246 (80.0%)	757 (7.3%)
HES APC	Lung cancer	33571	32287 (96.2%)	33571 (100%)	—	8585 (25.6%)
	Melanoma	11010	9384 (85.2%)	11010 (100%)	—	428 (3.9%)
	Renal cell cancer	8246	7965 (96.6%)	8246 (100%)	—	729 (8.8%)
SACT	Lung cancer	8742	8708 (99.6%)	8672 (99.2%)	8585 (98.2%)	—
	Melanoma	464	436 (94.0%)	453 (97.6%)	428 (92.2%)	—
	Renal cell cancer	772	764 (99.0%)	757 (98.1%)	729 (94.4%)	—
